# Effect of depression on sleep: Qualitative or quantitative?

**DOI:** 10.4103/0019-5545.49451

**Published:** 2009

**Authors:** Ravi Gupta, Sushant Dahiya, Manjeet Singh Bhatia

**Affiliations:** Department of Psychiatry, University College of Medical Sciences and GTB Hospital, Dilshad Garden, Delhi - 110 095, India

**Keywords:** Depression, sleep, severity of depression

## Abstract

**Background:**

The present study was designed to assess whether subjective sleep patterns differ between: (i) depressed patients and controls, and (ii) between subjects with different severity of depression. Based on available literature, it was hypothesized that sleep patterns must be different between the above mentioned groups.

**Materials and Methods:**

This study included 60 subjects with major depressive disorder and 40 subjects in the control group. Subjects with sleep disturbance secondary to any other factor, *e.g*., medical illness, environmental factors, other psychiatric illness etc, were not included in the study. Depression severity was assessed in the subjects with depression with the help of Beck Depression Inventory II. Subjective sleep complaints were noted in the presence of a reliable informant, preferably bed partner. All the information was recorded in a semistructured performa. Statistical analysis was done with the help of SPSS v 11.0. The Chi square and Fisher exact tests were used for categorical variables; independent t-test and one way ANOVA were applied for numerical variables. Ordinal variables were analyzed using Mann Whitney U and Kruskall-Wallis tests.

**Results:**

Depression and control groups were similar in age (*P* = 0.32) and gender (*P* = 0.14) distribution. Subjects in the depression group had lesser total sleep time (*P* = 0.001), longer sleep latency (*P* = 0.001), frequent awakenings (*P* = 0.04), greater wake-after-sleep onset and offset times (both *P* = 0.001), lesser sleep efficiency, and tended to wake up early (Mann Whitney U = 913.5; *P* = 0.05). Subjects with severe depression were different from the mild and moderate groups with regards to total sleep time (*P* = 0.002), night-time sleep (*P* = 0.007), and sleep efficiency (*P* = 0.001) even when the three groups were comparable in age.

**Conclusion:**

Depression is associated with sleep disturbances, not only qualitatively, but also quantitatively. Sleep disturbance arises only after a critical level of depression is reached, and depression of varying severity may selectively affect different sleep parameters.

## INTRODUCTION

Sleep disruption is a common feature of depression, manifesting itself as reduced total sleep time (TST), increase in sleep latency, and frequent awakenings during night-time sleep. Moreover, the sleep schedule usually changes during depression and, when subjectively asked, sleep is reported to be nonrestorative.[1]

Changes that occur in sleep during depression, may be confirmed objectively with the help of polysomnography. These studies demonstrate changes in sleep architecture, *e.g*., decrease in slow wave sleep (SWS) and REM latency, increase of REM density, and frequent arousals.[2] One study suggests that the severity of depression affects the polysomnographic findings as severely depressed subjects show lesser amount of stage two and three sleep, shortest REM latency, and shortest REM cycle duration than mild depression and control groups. On the contrary, stage 2 and 3 sleep is increased in mild depression as compared to controls.[3] Thus, evidence demonstrates that sleep architecture is not uniformly influenced by depression of varying severity.

Depression is a stressful situation and it leads to hyper-arousal, which is positively correlated with the severity of depression.[4] The cognitive theory of insomnia suggests that insomnia and hyperarousal run in a vicious cycle, where each exacerbates the other.[5] This is further substantiated by the fact that stresses that are accumulated during the day, show their effect on the sleep during night.[6] Neurobiological markers of stress, *e.g*., serum cortisol concentration, correlate with changes in sleep akin to those seen in depression.[7] Taken together, these proofs suggest that depression severity influences sleep architecture differently. In other words, depression of varying severity may not affect sleep patterns uniformly.

Although a lot of research has been dedicated to the neurobiology of insomnia in depression,[1,2,8–10] very little literature is available correlating the severity of depression with sleep changes. Moreover, whatever scant literature is available, that too, provides confounding results.[2,3]

The method of assessment of sleep is of paramount importance and it may affect the outcomes of the study. Various surveys conducted for the assessment of sleep patterns and disorders have used self-reported questionnaires with good sensitivity and specificity.[11–13] It has been reported that sleep history questionnaires could be reliable and valid measures of sleep timing, including time to bed, wake-up time, WASO, and sleep latency.[14] It also correlates well with traditionally used sleep diaries.[14] Wolfson *et al,*[15] conducted a survey and found that total sleep time and wake time reported by self-assessment questionnaire were no different from sleep amount assessed by well-known methods such as the use of a sleep diary or actigraphy. A recent study has shown that even insomnia sufferers can estimate the time reliably.[16] The information regarding sleep timing provided by them is reliable for clinical purposes and their subjective estimation is comparable to other measures.[16] Moreover, another study suggested a good correlation between subjective and objective sleep perception in depressed subjects.[17] In addition to polysomnographic findings, subjective perception of sleep correlates well with the underlying neurobiological processes. One study described the positive correlation of subjective and objective wake-after-sleep-onset (WASO), not only with each other, but also with the metabolic activity in the pontine tegmentum, thalamus, and cortical regions.[10] Hence, self-reports can be used to measure sleep in depressed patients.

It must be remembered that subjective impairment in sleep is associated with cognitive arousal and it perpetuates the insomnia further. Clinically, subjective perception of sleep is more important as it can influence the response to treatment.[18–19] Another interesting fact is that treatment of insomnia in depressed patients leads to better recovery of depression.[19]

Hence, it is worthwhile to study whether subjective sleep disturbances vary with the severity of depression, as it may guide us regarding the pathophysiology of depression and help in better management of the patients.

## MATERIALS AND METHODS

This study was conducted in the outpatient department of a medical college hospital. The objective of the study was explained to the subjects and their written informed consent was taken.

Subjects presenting with complaints of Major Depressive Disorder according to DSM-IV-TR criteria[20] were included in the study. However, subjects having any other medical disorder that may interfere with sleep, shift workers, subjects having other psychiatric disorders, substance abusers, treatment-resistant depression, environment-induced sleep problems, and those with history of specific recent stressors, *e.g*., recent death of a beloved, loss of job etc were excluded from the study. Patients taking sedating antidepressants for more than one week or who were prescribed antidepressants with hypnotic effects or benzodiazepine, those having history suggestive of sleep-related breath disorders or parasomnia were also excluded. Absence of a reliable informant to endorse sleep patterns of the study subjects led to the exclusion of the particular subject, but these informants were not necessarily the bed-partners of the patients included in the study.

Age- and sex-matched people who were accompanying the patients were screened to be included in the control group. They were also screened for the presence of major depressive disorder and for the exclusion criteria mentioned above.

Severity of depression was assessed with the help of Beck's Depression Inventory-II (BDI) and the depression group was divided into minimal, mild, moderate, and severe categories.[21] Sleep assessment was based on the information provided by the subjects. In routine practice, it is difficult to subject every patient of a sleep disorder to polysomnography and history is a reliable source of information in all such circumstances. Hence, in the present study, a cross-sectional assessment of sleep was done using a questionnaire.

Subjects with depression were asked to provide information regarding their usual sleep habits prior to the and following illness, and we compared them to exclude any recall bias as far as possible. Subjects in the control group were encouraged to provide information about the past three months.

Sleep history included total time spent in sleep during a day (twenty four hours), night-time spent on bed, time to bed, sleep latency, nocturnal awakenings, wake time after sleep onset, wake up time, wake time after sleep offset, refreshing sleep etc. They were asked if they frequently felt morning headaches or were sleepy during the day. As daytime napping is common in Asian cultures, data regarding daytime napping, its frequency, duration, time spent in naps, and dreaming during the naps were also gathered.

Statistical analysis was done with the help of SPSS v 11.0 for Windows. Descriptive analysis was done for all the variables. Categorical variables were analyzed with the help of Chi-Square or Fisher's Exact tests. Independent sample t-test was used to compare numerical data between depressed and nondepressed groups and one-way ANOVA with post-hoc analysis was applied in the subgroup analysis of the depression group. For the comparison of rank-order data, Mann-Whitney U and Kruskall- Wallis tests were used.

## RESULTS

Sixty subjects with major depressive disorder and 40 control subjects were included in the present study. Both groups were similar in gender distribution (control group: 65% male; Cases = 73% male; *P* = 0.14); age (control group = 20.87 ± 8.01; Cases = 31.86 ± 10.97; *P* = 0.32), frequency of not following the sleep schedule (*P* = 0.1), frequency of awakenings per night (*P* = 0.67), morning headache (*P* = 0.08), abnormal movements during sleep (*P* = 0.34), habit of daytime napping (*P* = 0.37), number of naps per day (*P* = 0.48), total time spent in naps (*P* = 0.07), and whether naps were planned or not (*P* = 0.59).

A significantly higher number of depressed subjects complained of nocturnal awakenings (*P* = 0.009), nonrefreshing sleep (*P* = 0.001), and difficulty in leaving bed in the morning (*P* = 0.01) than the controls. On the other hand, daytime sleep (*P* = 0.001) was more frequent in the control group. Stressful periods changed the sleep pattern of the control group more commonly as compared to the depressed group (*P* = 0.01). Other variables are shown in Table 1. Although the time to go to bed was not different between the groups, time to wake up was significantly different (Mann Whitney U = 913.5; *P* = 0.05) and it was seen that depressed subjects tended to wake up earlier.

**Table 1:** Sleep-related variables in depression and control groups

Variable	Depression	*N*	Mean	SD	SE	Sig
Total sleep time (H)	Yes	60	5.90	2.35	0.30	
	No	40	7.73	1.41	0.22	0.001
Sleep latency (Min)*	Yes	59	34.83	28.57	3.71	
	No	40	10.12	8.20	1.29	0.001
Duration of nighttime sleep (H)*	Yes	59	5.74	2.24	0.29	
	No	40	7.18	1.33	0.21	0.001
Frequency of night awakenings/Week†	Yes	25	5.92	1.18	0.23	
	No	7	4.85	1.06	0.40	0.04
Awaken after… hours at night†	Yes	25	1.82	0.85	0.17	
	No	7	3.78	1.67	0.63	0.02
WASO (min)†*	Yes	24	41.45	18.38	3.75	
	No	7	6.42	5.56	2.10	0.001
WASF (min)*	Yes	59	19.08	22.34	2.90	
	No	40	7.00	6.96	1.10	0.001
Sleep efficiency (%)*	Yes	59	74.99	24.7	3.21	
	No	40	95.6	3.38	0.53	0.001
Frequency of naps/Week†	Yes	15	6.33	0.97	0.25	
	No	12	5.50	0.67	0.19	0.01
Beck's score	Yes	60	20.85	6.58	0.85	
	No	40	5.60	2.78	0.43	0.001

Independent sample t-tests;

* Details not available for one depressed subject; excluded from the analysis;

† Those without this complaint were excluded from the analysis

**Figure 1:**
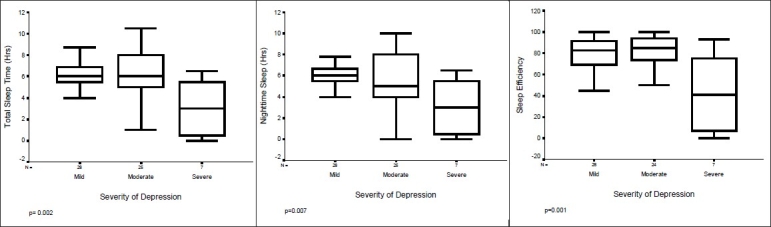
Sleep variables in relation to severity of depression. Post-hoc Tukey shows severe group to be different from the other two groups at *P* = 0.05

When the depression group was further categorized according to the severity of depression into ‘mild’, ‘moderate,’ and ‘severe’ categories, it was found that most of the subjects had mild to moderate depression and severe depression was seen in only 16% of the subjects. The age was not different among these three groups (F = 0.23; *P* = 0.977). Severity of depression did not affect any of the categorical variables. Time to bed and wake up time also were not different among these groups (Kruskal Wallis test; *P* = 0.79 and 0.86 respectively). However, a few other parameters, *e.g*., total sleep time, night-time sleep, sleep efficiency, frequency of nocturnal awakenings (days/week), and time of first awakening after night-time sleep were different between the three groups [Table 2 and Figure 1].

**Table 2:** Sleep parameters among groups based on severity of depression

Variable	Severity	*N*	Mean	SD	SE	95% CI		Sig.
							
						Lower	Upper	
Frequency of night awake/Week†	Mild	20	6.50	0.90	0.26	5.92	7.07	0.02
	Moderate	25	5.54	1.21	0.36	4.73	6.36	
	Severe	7	4.50	0.70	0.50	−1.85	10.85	
Awakening hours after sleep#	Mild	20	2.20	0.78	0.22	1.71	2.70	0.05
	Moderate	25	1.36	0.80	0.24	0.82	1.90	
	Severe	7	2.00	0.00	0.00	2.00	2.00	

One-Way ANOVA;

† On post-hoc Tukey, three groups were not found to bedifferent from each other;

# Post-hoc Tukey shows the moderate group to be different from the mild group at *P* = 0.05

## DISCUSSION

In this study, almost all sleep variables were affected by the presence of depression. Briefly, depressed subjects had lesser total as well as nocturnal sleep times, longer sleep latency, higher prevalence of nocturnal awakenings, early awakening after getting sleep, took more time to sleep again, had increased frequency of night awakenings, and took more time to leave their beds in the morning. Similarly, the majority of them complained of nonrefreshing sleep and had lesser sleep efficiency. Interestingly, probably for the first time, it has been shown that depression has not only the qualitative but also the quantitative impact on subjective sleep parameters, even when the three groups were comparable with regards to confounding variables including age.

The differences between the objective sleep parameters of depressed subjects and the control group have been a matter of extensive research and this study corroborates previous results. Existing literature describes that total sleep time is reduced and sleep latency is increased in most psychiatric disorders, including depression.[22] Another study reported that subjective sleep parameters, especially, sleep latency, perceived number of awakenings, and WASO were similar between depression cases and controls, despite the fact the subjects were moderately depressed and had significant higher scores on HRSD and BDI than the controls.[9] They proposed that sleep changes may only be seen when the depression is more severe, in other words, depression may have a flooring effect on the sleep disturbance. Another study demonstrated that BDI scores were positively correlated with the frequency of night awakenings and higher in subjects with combined insomnia (initial and middle). In other words, where values of sleep latency and WASO were more than 31 minutes per night, frequency of nocturnal awakenings was found to be correlated with the severity of depression in the present study also.[23]

Although sleep latency and WASO were significantly different between the depressed cases and the controls, this effect did not persist when depression subjects were further grouped according to the severity of depression, contrary to the previous literature. However, the differences could be secondary to the differences in the diagnosis of depression, as we have included subjects with clinically significant depression and the severity was assessed with BDI. On the other hand, in the study by Taylor *et al*,[23] all subjects were analyzed with BDI, irrespective of clinical diagnosis of depressed mood. Secondly, insomnia subjects were older in that study as compared to our study population, and this could be confounding factor. Thirdly, it is possible that depression affects a few sleep parameters qualitatively, others quantitatively, and has a mixed affect on some of them. At present, our knowledge in this area is limited and it demands further study.

However, this study had a few methodological limitations, *e.g*., the sample size was small due to stringent inclusion and exclusion criteria. This also prevents us from generalizing the results of the study, especially in subjects with co-morbid medical or psychiatric disorders. Secondly, we did not assess the effect of the number of depressive episodes, the duration of the current episode, or the effect of life stressors on the sleep variables. Although one recent study has shown that these parameters do not correlate with polysomnographic findings in depressed patients,[24] we still propose that more research is required. Thirdly, the sample size in the severe depression group is very small and hence, results should be interpreted with caution.

In conclusion, this is probably the first study to demonstrate that depression has not only qualitative but also quantitative effects on subjective sleep disturbances. Further studies are required to find out whether objective evidence also exists for the same and to determine the neurobiological underpinnings of these findings.
